# DAGMNet: Dual-Branch Attention-Pruned Graph Neural Network for Multimodal sMRI and fMRI Fusion in Autism Prediction

**DOI:** 10.3390/biomedicines13092168

**Published:** 2025-09-05

**Authors:** Lanlan Wang, Xinyu Li, Jialu Yuan, Yinghao Chen

**Affiliations:** 1 School of Computer Science and Engineering, Central South University, Changsha 410083, China; 224701046@csu.edu.cn; 2 School of Electrical Engineering, Xi’an Jiaotong University, Xi’an 710049, China; jialuyuan@stu.xjtu.edu.cn; 3 School of Mathematics and Statistics, Central South University, Changsha 410083, China; chenyinghao1@csu.edu.cn

**Keywords:** autism spectrum disorder, graph neural network, multimodal, dual-branch, attention-pruned

## Abstract

**Background**: Accurate and early diagnosis of autism spectrum disorder (ASD) is essential for timely intervention. Structural magnetic resonance imaging (sMRI) and functional magnetic resonance imaging (fMRI) provide complementary insights into brain structure and function. Most deep learning approaches rely on a single modality, limiting their ability to capture cross-modal relationships. **Methods**: We propose DAGMNet, a dual-branch attention-pruned graph neural network for ASD prediction that integrates sMRI, fMRI, and phenotypic data. The framework employs modality-specific feature extraction to preserve unique structural and functional characteristics, an attention-based cross-modal fusion module to model inter-modality complementarity, and a phenotype-pruned dynamic graph learning module with adaptive graph construction for personalized diagnosis. **Results**: Evaluated on the ABIDE-I dataset, DAGMNet achieves an accuracy of 91.59% and an AUC of 96.80%, outperforming several state-of-the-art baselines. To validate the method’s generalizability, we also validate it on ADNI datasets from other degenerative diseases and achieve good results. **Conclusions**: By effectively fusing multimodal neuroimaging and phenotypic information, DAGMNet enhances cross-modal representation learning and improves diagnostic accuracy. To further assist clinical decision making, we conduct biomarker detection analysis to provide region-level explanations of our model’s decisions.

## 1. Introduction

Autism spectrum disorder (ASD) is a prevalent neurodevelopmental condition characterized by deficits in social communication and the presence of restrictive and repetitive behaviors. According to the Centers for Disease Control and Prevention (CDC), approximately 1 in 36 children in the United States is diagnosed with ASD [1]. Beyond its early developmental onset, ASD is often accompanied by psychiatric comorbidities such as anxiety and depression [2], complicating both diagnosis and intervention. Clinical diagnostic tools such as the Autism Diagnostic Observation Schedule (ADOS) and the Autism Diagnostic Interview-Revised (ADI-R) [3,4] remain the gold standard; however, their reliance on subjective behavioral assessments can result in variability, delayed detection, and limited scalability.

Advances in neuroimaging have opened new avenues for ASD research. Structural magnetic resonance imaging (sMRI) enables the analysis of cortical and subcortical anatomy [5]. In contrast, functional magnetic resonance imaging (fMRI) reveals brain activity patterns and connectivity via blood oxygen level-dependent (BOLD) signals. Early machine learning studies using handcrafted features such as cortical thickness and voxel-based morphometry [6] demonstrated moderate diagnostic accuracy. More recently, deep learning has shown great promise in automatically learning discriminative features from raw MRI data [7,8,9].

Multimodal approaches combining sMRI and fMRI have emerged to exploit their complementary information [10,11]. For example, attention-based fusion strategies [12] and adversarial alignment [13] have been proposed to improve cross-modal consistency. However, many existing frameworks still rely on either early or late fusion heuristics, which may not fully capture complex inter-modality relationships. Graph neural networks (GNNs) have recently become a powerful paradigm for modeling brain connectivity in both individual and population-level graphs [14,15]. Variants such as BrainGNN [16] incorporate attention-based pooling and spatial-temporal priors, while other works have introduced dynamic graph construction [17] or hierarchical multi-atlas learning. Despite these advances, three major limitations remain: (1) Most existing studies focus on a single modality, missing cross-modal synergies between sMRI and fMRI. (2) Many graph-based methods employ static atlas-based or similarity-based graphs, limiting their ability to capture subject-specific topological variations. (3) Dense and static population graphs are prone to over-smoothing and overfitting, especially in the presence of noise or redundant connections.

Our main contributions are as follows:

1. We utilize both sMRI and fMRI data, incorporating three commonly used fMRI-based brain atlases (HO, AAL, and EZ), and design modality-specific feature extraction strategies. These methods effectively capture essential features from three-dimensional anatomical structures and topological representations of brain regions, leading to an optimal and discriminative multimodal feature set.
2. We design a multimodal aware representation learning module that separately learns shared and modality-specific features from structural and functional inputs. These components are adaptively fused via a gating mechanism to generate a compact and informative feature representation, which serves as the individualized embedding for each subject.
3. We design a phenotypic similarity-based population graph and apply a dynamic attention-guided pruning strategy to optimize subject connectivity and improve prediction performance iteratively.
4. Experiments on the ABIDE-I dataset demonstrate the superiority of DAGMNet, achieving an accuracy of 91.59% and an AUC of 96.80%, outperforming several state-of-the-art models. Extensive ablation studies and t-SNE visualizations further support the interpretability and robustness of our framework.

## 2. Materials and Methods

### 2.1. Method Overview

**Figure 1:**
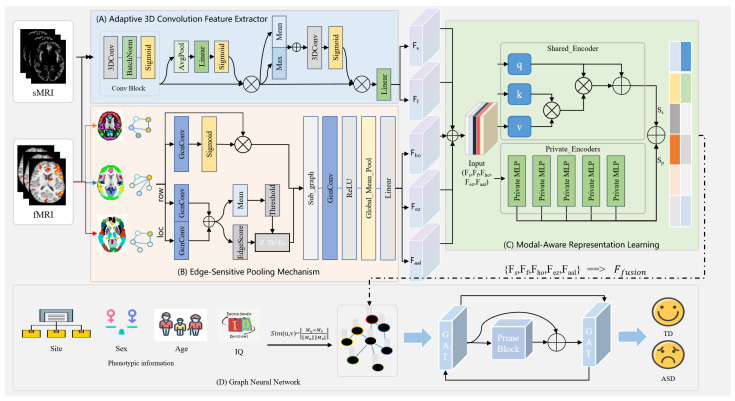
Overall framework of the proposed DAGMNet for predicting ASD using multimodal data.

As shown in Figure 1, the proposed DAGMNet framework is mainly composed of four components: (A) an adaptive 3D convolution feature extractor for sMRI and fMRI, (B) an edge-sensitive pooling mechanism (ESPM) for fMRI, (C) a modal-aware representation learning module, and (D) a phenotype-guided population graph learning module. Specifically, sMRI data are processed through a series of adaptive 3D convolution blocks to extract structural features at the subject level, in which the dual-branch design uses both average-pooling and maximum-pooling signals to enhance feature expression capabilities. fMRI data are first converted from multiple spatial perspectives to graph representations, and refined functional connection patterns are extracted through a multi-stage graph convolutional network with edge pruning and edge-sensitive pooling. The features extracted from the two modalities are input into the modal-aware representation learning module, which contains a shared encoder and multiple private encoders to separate cross-modal shared representations from modal-specific representations.

This design enables better feature alignment and retains complementary features of sMRI and fMRI. To model relationships between subjects, a population map is constructed based on phenotypic similarities (such as study location, gender, age, and IQ). The graph attention network (GAT) distributes information between nodes while utilizing pruning mechanisms to suppress irrelevant connections selectively. The final node embedding is used for the prediction of ASD.

### 2.2. Feature Extraction

To comprehensively model both structural and functional neuroimaging modalities, DAGMNet employs two parallel branches for sMRI and fMRI, respectively. These branches aim to extract features, leverage three-dimensional convolution, and graph learning methods.

#### 2.2.1. sMRI and fMRI Image Feature Extraction

To fully capture the structural and functional features contained in sMRI and fMRI data, we propose an Adaptive Convolutional Feature Extractor (ACFE) module, which combines 3D convolutional operations with channel-wise and spatial attention mechanisms. The input sMRI and fMRI data are denoted as $F_{s},\;F_{f}$, where *B* is the batch size, and D, H, W represent depth, height, and width dimensions, respectively. The ACFE module uses 3D convolutional layers to extract preliminary volume features. The channel’s attention mechanism adaptively fuses these features. Specifically, given a feature map *F*, global average pooling is performed to summarize spatial information into a channel descriptor vector $z_{c}$:

(1) $$z_{c}=\frac{1}{D^{\prime }H^{\prime }W^{\prime }}\sum_{i,j,k}F_{c,i,j,k},$$

where $z_{c}$ denotes the descriptor for channel *c*. Two fully connected layers with a ReLU activation followed by a sigmoid function σ generate channel attention weights *s*:

(2) $$s=\sigma \left(W_{2}\cdot \mathrm{ReLU}\left(W_{1}\cdot z\right)\right),$$

where $W_{1}$, $W_{2}$, and *r* are the reduction ratio controlling model complexity. The original feature maps are modulated channel-wise by these weights to yield $\tilde{F}$. A spatial attention mechanism further emphasizes salient spatial regions within $\tilde{F}$. Channel-wise aggregation is first performed via average pooling ($F_{\mathrm{avg}}$) and max pooling ($F_{\max}$). The aggregated maps are concatenated and processed by a 3D convolution layer to produce a spatial attention map, $M_{s}$, defined as:

(3) $$M_{s}=\sigma \left(\mathrm{Conv}3D(\left[F_{\mathrm{avg}};\;F_{\max}\right])\right),$$

where the spatially modulated feature maps are obtained by element-wise multiplication of $\tilde{F}$ and $M_{s}$. After processing through multiple stacked convolutional and ACFE layers, adaptive average pooling is used to obtain fixed-size feature maps, which are flattened into a global feature vector *f*. A fully connected layer further projects *f* into a final global representation $f_{\mathrm{global}}$:

(4) $$f_{\mathrm{global}}=\mathrm{ReLU}\left(Wf+b\right),$$

where *W* and *b* are learnable parameters. Consequently, the global feature vectors derived from sMRI and fMRI inputs through the ACFE module are defined as:

(5) $$F_{s}=\phi_{\mathrm{ACFE}}\left(F_{s}\right),F_{f}=\phi_{\mathrm{ACFE}}\left(F_{f}\right).$$

By leveraging this adaptive feature extraction process, the ACFE module effectively highlights both channel-specific attributes and spatially salient patterns within brain imaging data, facilitating robust multimodal representation learning.

#### 2.2.2. Multi-Atlas Brain Graph Feature Extraction

We adopt the method proposed by Pan et al. [18] to represent brain imaging data as a graph. Specifically, for the Harvard–Oxford (HO) atlas, fMRI data are conceptualized as a graph structure, where nodes correspond to different brain regions and edges signify functional connections between these regions. Following the construction method outlined by Huang et al. [19], each of the fifty-five regions in the right hemisphere is connected to its corresponding region in the left hemisphere, and vice versa. Additionally, to mitigate potential inaccuracies arising from data collected using different devices, all brain regions are linked to the average time series. The original graph is denoted as $G_{i}={\left\{V_{i},A_{j}\right\}}_{\left(i=1\right)}^{N}$, where *A* represents the adjacency matrix of *G*, $V=\{V_{1},V_{2},\ldots ,V_{M}\}$ denotes the set of nodes in *G*, and *N* and *M* denote the number of subjects and brain regions, respectively. In summary, the HO atlas consists of 111 nodes and 6270 edges, the EZ atlas comprises 116 nodes and 7560 edges, and the Anatomical Automatic Labeling (AAL) atlas encompasses 116 nodes and 7560 edges.

To extract consistent graph representations across heterogeneous brain atlases, we design an edge-sensitive pooling mechanism that adaptively reweights node features and prunes weak connections while preserving all nodes. Each subject’s brain network is represented as an undirected graph G=(X,A), where *X* denotes the node feature matrix and *A* the adjacency matrix.

The edge-sensitive pooling mechanism computes node embeddings by applying spectral graph convolution with self-loops added to the adjacency matrix. The degree matrix is normalized accordingly, and a learnable weight matrix is used to transform the input features, followed by a ReLU activation. The node importance coefficients are denoted as α. A single-layer affine transformation and a sigmoid activation are applied to the node embeddings. These coefficients are used to recalibrate the node features via element-wise multiplication along the nodes, producing updated node features $X^{\prime }$.

(6) $$\alpha =\sigma \left(Hw\right),\;\;\;X^{\prime }=X⊙\alpha ,$$

where *w* is a weight vector, σ(·) denotes the sigmoid activation, and ⊙ denotes element-wise multiplication along nodes. Edge scores $e_{ij}$ are defined as the sum of weights of connected nodes, reflecting edge strength:

(7) $$e_{ij}=\alpha_{i}+\alpha_{j}.$$

The edge strength between two nodes is determined by summing the corresponding node importance coefficients. This results in edge scores that reflect the combined significance of the connected nodes and guide the subsequent edge pruning process. Edges are pruned by retaining only those whose scores exceed the mean of all edge scores, resulting in a sparsified adjacency matrix. Graph convolution is subsequently performed on the sparsified graph, and global mean pooling is applied to extract the final graph representation. This process is independently applied to HO, EZ, and AAL brain atlases to yield consistent graph embeddings as $F_{ho}$, $F_{ez}$, and $F_{aal}$.

### 2.3. Multimodal Aware Representation Learning

To effectively fusion heterogeneous neuroimaging information, we propose a unified module termed Multimodal Aware Representation Learning (MARL), as shown in Figure 1C, which jointly models both shared and modality-specific components across structural and functional views. The multimodal input consists of features from sMRI, fMRI, and graph-based embeddings derived from multiple brain atlases:

(8) $$F=\{F_{s},F_{f},F_{ho},F_{ez},F_{aal}\},$$

where $F_{s},F_{f}$ denote features extracted from sMRI and fMRI, respectively; and $F_{ho},F_{ez},F_{aal}$ are graph embeddings obtained from HO, EZ, and AAL atlases. *B* represents the batch size and *d* is the feature dimension per modality. These modality-specific features are concatenated along the feature dimension to form a unified representation:

(9) $$X=[F_{s},F_{f},F_{ho},F_{ez},F_{aal}],$$

where [·] denotes concatenation. Each modality feature $F_{m}$ is separately processed by two parallel encoders: a shared encoder $E_{s}\left(\cdot \right)$ that extracts modality-invariant representations $s_{m}$, and a private encoder $E_{p}^{\left(m\right)}\left(\cdot \right)$ that captures modality-specific information $p_{m}$. To balance the contributions of shared and private components, a gating mechanism computes adaptive weights $g_{m}\in \left[0,1\right]$ by projecting the concatenated embeddings $\left[s_{m};p_{m}\right]$ through a sigmoid function. The fused representation for each modality is obtained by element-wise weighting and summation:

(10) $$f_{m}=g_{m}⊙s_{m}+\left(1-g_{m}\right)⊙p_{m},$$

where ⊙ denotes element-wise multiplication. This design allows adaptive fusion between shared and modality-specific signals.

Finally, all fused modality features ${\left\{f_{m}\right\}}_{m=1}^{5}$ are concatenated and passed through a fully connected layer ϕ(·) with ReLU activation to produce a compact latent embedding:

(11) $$F_{\mathrm{fusion}}=\phi \left(\left[f_{1},f_{2},\ldots ,f_{5}\right]\right),$$

where the fused representation captures both consistent semantics shared across modalities and individualized modality-specific variations, thus facilitating downstream classification and prediction tasks.

### 2.4. Graph Neural Networks

Population graph construction is based on phenotypic similarity, as shown in Figure 1D. We construct a population graph G=(V,E), where each node $v_{i}\in V$ corresponds to a subject, and edges E represent phenotypic similarity between subjects. Each node is associated with a subject-level feature vector $x_{i}$, obtained from the multimodal fusion module described previously. The cosine similarity between node feature vectors determines edge connectivity:

(12) $$\mathrm{Sim}\left(u,v\right)=\left|\frac{x_{u}^{⊤}x_{v}}{∥x_{u}∥∥x_{v}∥}\right|,$$

where ∥·∥ denotes the Euclidean norm and $\left|\cdot \right|$ denotes the absolute value, ensuring non-negative similarity scores. The adjacency matrix *A* is binarized by thresholding the similarity scores at a predefined value th:

(13) $$A\left(u,v\right)=\left\{\begin{array}{cc} 1, & \mathrm{if}\;\mathrm{Sim}(u,v)>\mathrm{th},\\ 0, & \mathrm{otherwise}, \end{array}\right.$$

where the process results in an undirected graph with 871 nodes, corresponding to the total number of subjects included in the dataset.

Dynamic attention-based graph pruning alleviates the common over-smoothing problem in depth map networks. We introduce a Dynamic Attention-based Graph Pruning (DAGP) mechanism, which adaptively prunes low-confidence edges during training. The DAGP is built based on the GAT, where attention coefficients $\alpha_{ij}$ measure the importance of edges based on node features $x_{i}$. Specifically, node features are linearly transformed, and pairwise attention scores are computed via a shared attention mechanism followed by a LeakyReLU activation and normalized using a softmax over neighbors N(i). The updated node embeddings $z_{i}$ are aggregated as a weighted sum of neighboring transformed features.

Formally, the attention score $\alpha_{ij}$ quantifies the relevance of the edge between nodes *i* and *j*, enabling the model to focus on informative connections. To refine graph topology and reduce noise, only the top p% edges with the highest attention weights are retained, producing a pruned edge set $E^{\prime }$ and corresponding graph $G^{\prime }=\left(V,E^{\prime }\right)$. A subsequent GAT layer operates on $G^{\prime }$ to yield the final node embeddings $z_{i}^{\left(2\right)}$.

The model outputs predicted probabilities ${\hat{y}}_{i}$ via a sigmoid activation on the final embeddings, and is optimized by minimizing a binary cross-entropy loss augmented with $L_{2}$ regularization on learnable parameters $\left\{W_{l}\right\}$:

(14) $$L=-\sum_{i=1}^{N}\left[y_{i}\log{\hat{y}}_{i}+\left(1-y_{i}\right)\log\left(1-{\hat{y}}_{i}\right)\right]+\lambda \sum_{l}{∥W_{l}∥}_{2}^{2},$$

where $y_{i}$ is the true binary label and λ controls the regularization strength.

### 2.5. Dataset

Our experiments use the Autism Brain Imaging Data Exchange (ABIDE-I), an open-access resource for multimodal neuroimaging data cited in [20]. Specifically, we use ABIDE-I, which compiles MRI data and comprehensive phenotypic information from 17 international sites. This study focuses on rs-fMRI data and the corresponding non-imaging phenotypic details. To ensure data integrity and method consistency, we employ the preprocessed version of the dataset available from the Preprocessed Connectomes Project. This preprocessing is performed using the Configurable Pipeline for the Analysis of Connectomes (CPAC). To ensure the highest data quality, we exclude subjects with incomplete time series, insufficient brain coverage, severe head movement, or other scan abnormalities. Following these exclusion criteria, the dataset ultimately used for analysis included 871 subjects, including 403 individuals diagnosed with autism spectrum disorders and 468 typically developing controls (TC). For the analysis phase, we employ the Harvard–Oxford (HO) cortical structural atlas, the EZ atlas, and the AAL atlas to delineate activation states across various brain regions. Additionally, phenotypic data play an important role in constructing the population graph structure for our study.

### 2.6. Implementation

We evaluate our proposed model on the widely used ABIDE-1 dataset containing sMRI and fMRI data from individuals with ASD and healthy controls. Following the standard pretreatment process, we extract brain regions based on multiple atlases (HO, EZ, and AAL) and construct structural and functional graphs for each subject. All experiments are performed on a workstation equipped with an NVIDIA RTX 3090 GPU (NVIDIA Corporation, Santa Clara, CA, USA) and 128 GB of RAM. The model is implemented using PyTorch Geometric (version 2.5.2). We integrate Monte Carlo dropout for uncertainty estimation into the DAGMNet framework. We use the Adam optimizer, the initial learning rate of $1\times 10^{-3}$, and a batch size of 32. The training objective is optimized using the binary cross-entropy loss function to predict binary class labels. We perform 10-fold cross-validation.

### 2.7. Competitive Methods and Evaluation Metrics

To validate the superiority of DAGMNet, we compare it with several ASD prediction methods. Specifically, Graph-based methods: We compare our model with several graph neural network approaches. Huang et al. [17] introduced adaptive graph learning using variational edge inference and Monte Carlo edge dropout to reduce over-smoothing in deep GNNs. Mahler et al. [21] proposed a Transformer framework with multi-atlas encoding and semi-supervised pretraining for fine-grained spatial learning. Heterogeneous feature fusion: Manikantan et al. [22] extracted radiomics and volumetric features as node attributes and modeled them via shallow GCNs. Zheng et al. [23] designed a dual-level GNN with ROI- and subject-level modules for hierarchical integration of imaging and phenotypic data. Multimodal fusion: Chen et al. [13] proposed an adversarial encoder for aligning modality-specific distributions, with modality-level weighting. Zhang et al. [12] introduced an attention-based fusion with adaptive graph learning. Wang et al. [24] learned population graphs in a latent similarity space. Li et al. [25] developed BPGLNet to jointly learn brain- and population-level topologies. We compare our method with several recent graph-based approaches for ASD diagnosis. Liu et al. [26] proposed a multimodal multi-kernel graph learning framework for improved ASD prediction and biomarker discovery. Shan et al. [27] developed MTGWNN, a multi-template graph wavelet network capturing multi-scale brain connectivity for ASD identification. Liu et al. [28] introduced DML-GNN, leveraging dual brain atlases and multi-feature learning to enhance diagnosis.

To evaluate the performance of our proposed model, we use several widely used evaluation metrics, including accuracy (ACC), sensitivity (SEN), specificity (SPE), and area under the receiver operating characteristic curve (AUC). All evaluation metrics are reported as percentages (%) for consistency. These metrics are computed based on the confusion matrix and are defined as follows:

(15) $$\mathrm{ACC}=\frac{TP+TN}{TP+TN+FP+FN}\times 100,$$

(16) $$\mathrm{SEN}=\frac{TP}{TP+FN}\times 100,$$

(17) $$\mathrm{SPE}=\frac{TN}{TN+FP}\times 100,$$

(18) $$\mathrm{AUC}=\int_{0}^{1}\mathrm{TPR}d\left(\mathrm{FPR}\right)\times 100,$$

(19) $$\mathrm{TPR}=\frac{TP}{TP+FN},\;\;\;\mathrm{FPR}=\frac{FP}{FP+TN}.$$

where TP, TN, FP, FN, TPR, and FPR denote true positives, true negatives, false positives, false negatives, true positive rate, and false positive rate, respectively.

## 3. Results

### 3.1. Comparison with Other Methods

**Figure 2:**
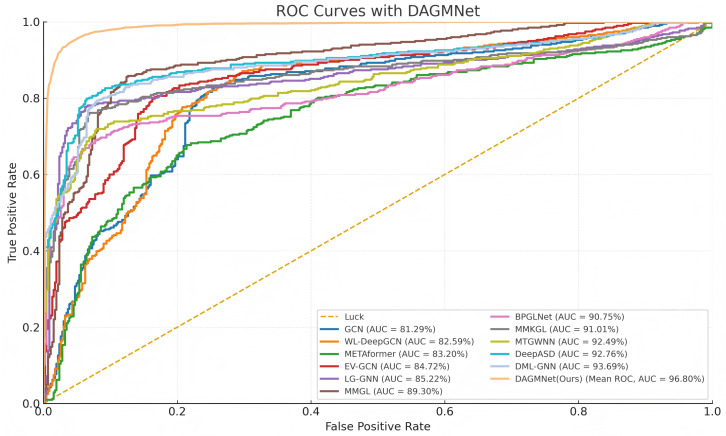
ROC Comparison of DAGMNet and Baseline Methods.

As shown in Table 1, DAGMNet performs better than several state-of-the-art ASD prediction methods, including unimodal, multimodal, and graph-based models. Specifically, DAGMNet achieves the highest accuracy of 91.59% , outperforming BPGNet (90.93%) and MMGL (89.77%), which reflects its enhanced overall classification capability. In terms of sensitivity, DAGMNet achieved 90.33%, highlighting its effectiveness in correctly identifying patients with ASD. Moreover, DAGMNet achieved the highest specificity of 93.23%, exceeding all baseline models and showing a robust performance in identifying non-ASD individuals. Although DeepASD and BPGNet report slightly higher AUC scores, DAGMNet still achieves a competitive AUC of 96.80%, indicating balanced discriminant power across categories. These results highlight the advantages of our dual-branch representation and attention-based graph pruning fusion strategy, especially in their ability to fuse structural, functional, and phenotypic features to enable accurate and personalized ASD diagnosis. As shown in Figure 2, our proposed DAGMNet consistently outperforms all competing state-of-the-art methods across the entire FPR range. Specifically, DAGMNet achieves the highest sensitivity and specificity, indicating superior capability in accurately distinguishing ASD patients from controls. Moreover, its AUC value approaches 97%, highlighting the robustness and strong generalization ability of our model.

### 3.2. Ablation Study

#### 3.2.1. Impact of Key Architecture Components

**Figure 3:**
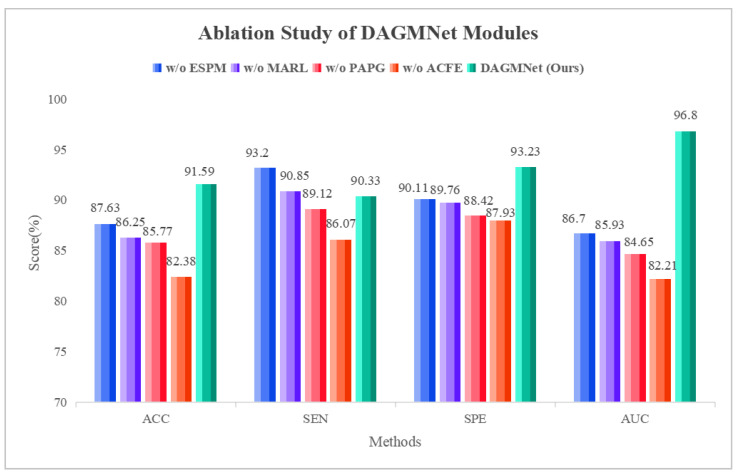
Ablation study on the contribution of DAGMNNet modules.

To validate the effectiveness of each module within DAGMNet, we conduct a series of ablation experiments by selectively removing key components: the edge-sensitive pooling (ESPM), the multimodal aware representation learning (MARL), the phenotype aware population graph (PAPG), and the sMRI feature branch (ACFE). As shown in Table 2, removing the edge-sensitive pooling (w/o ESPM) causes a noticeable drop in AUC (from 96.80% to 86.70%), indicating that adaptive pruning and edge-weighted pooling are crucial for extracting informative graph features from fMRI. Eliminating the fusion module (w/o MARL) further degrades performance, reducing the accuracy to 86.25%, confirming that cross-modal interactions learned via shared private encoders are essential for robust representation learning. When the phenotype-aware graph neural network is removed (w/o PAPG), accuracy declined by nearly 6%, highlighting the benefit of incorporating demographic factors such as age, sex, site, and IQ to model population-level subject similarity. Lastly, removing the sMRI branch (w/o ACFE) yielded the worst performance (AUC = 82.21%), emphasizing the complementary role of structural features in ASD diagnosis. These results confirm that each component of DAGMNet plays a vital role in achieving state-of-the-art performance. As shown in Figure 3, the ablation study demonstrates that removing any module degrades performance, while the complete DAGMNet consistently achieves the best results. In particular, the ACFE module proves essential, as its removal leads to the largest drop across all metrics (ACC, SEN, SPE, and AUC). These results confirm that each component contributes to the final performance, with ACFE playing the most critical role.

**Figure 4:**
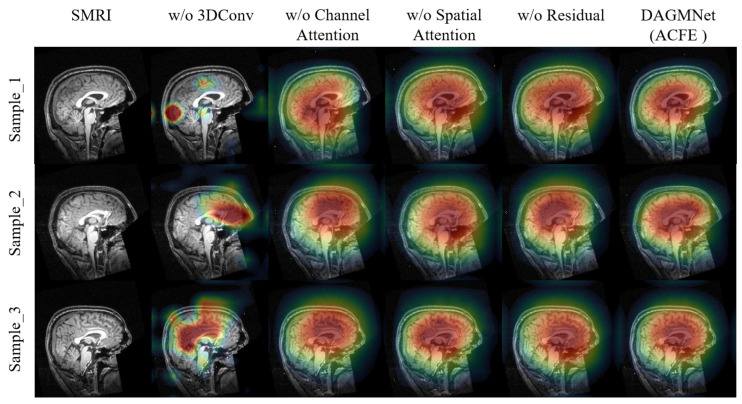
Visualization of the ablation study on the ACFE module using Grad-CAM.The colors represent the intensity of the model’s focus, where red indicates the most influential regions, yellow and green represent moderate influence, and blue shows areas with minimal contribution to the model’s decision.

To assess the contribution of each sub-component in ACFE, we conduct both qualitative and quantitative ablation studies. As shown in Figure 4, removing any module leads to less focused and less interpretable activation maps. In contrast, the full ACFE consistently highlights concentrated and meaningful brain regions, which are highly relevant to ASD prediction. As shown in Table 3, the quantitative results further validate these findings. The full ACFE model achieves the highest performance (ACC = 91.59%, SEN = 90.33%, SPE = 93.23%, AUC = 96.80%). Removing 3D convolution results in the largest performance degradation (ACC = 83.20%, AUC = 84.00%), highlighting its essential role in capturing 3D structural information. Removing channel or spatial attention also leads to notable drops in AUC (≈89%), confirming the effectiveness of attention mechanisms in enhancing feature discriminability. The residual connection contributes moderately, improving stability and generalization.

#### 3.2.2. Impact of Different Modality Inputs

We validate the effectiveness of different input modalities on model performance, including sMRI, fMRI, and multi-atlas data. As shown in Table 4, using only sMRI or fMRI results in relatively lower accuracy and AUC. Notably, introducing multi-atlas fMRI graphs leads to a significant performance boost, demonstrating the benefit of incorporating diverse functional parcellations. Our proposed DAGMNet model, which fuses both structural and multi-source functional features, achieves the best results. This highlights the importance of comprehensive multimodal fusion in capturing complementary anatomical and functional patterns for ASD prediction. The DAGMNet integrates atlas-free image streams with multi-atlas graph inputs via modality-aware fusion and dynamic graph learning, further increasing specificity while maintaining maximal AUC. This confirms that the proposed design reduces dependence on predefined atlases through two complementary mechanisms: inclusion of atlas-independent feature pathways and use of a diverse atlas ensemble to stabilize graph representations.

**Figure 5:**
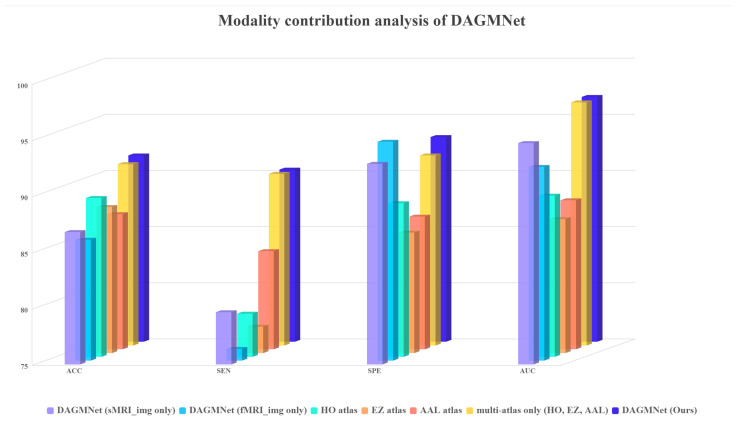
Modality contribution analysis of DAGMNNet.

We further evaluate the influence of different input modality settings. As shown in Figure 5, our modality ablation study demonstrates that using only sMRI or fMRI significantly reduces performance, while incorporating single-atlas information achieves better but still suboptimal results. The combination of multiple atlases improves performance further, and the full DAGMNet with multi-modality and multi-atlas inputs consistently achieves the highest scores across all metrics. These results confirm the necessity of integrating both modalities and atlas-based features for optimal ASD prediction.

#### 3.2.3. Impact of Feature Fusion Mechanisms

We validate the effectiveness of our MARL fusion strategy by comparing it with several widely used alternatives, including simple concatenation, self-attention, efficient cross-modal attention (ECMA), and cross-attention. As shown in Table 5, our fusion design consistently outperforms all baselines across accuracy, sensitivity, specificity, and AUC. In particular, the performance gap between DAGMNet and concatenation or ECMA underscores the importance of modeling both shared and modality-specific features. These results validate that our attention-guided gating mechanism enables more effective integration of complementary multimodal cues.

### 3.3. ROC Curve Analysis

**Figure 6:**
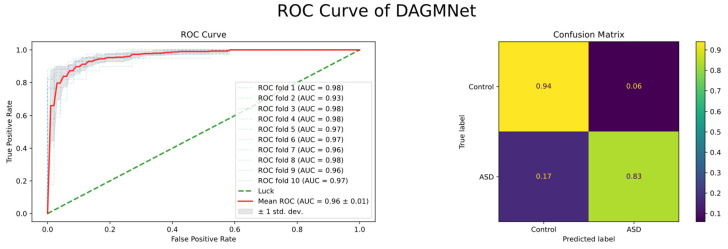
Receiver Operating Characteristic (ROC) curves of DAGMNet and baseline models. DAGMNet achieves the highest AUC, indicating superior prediction performance between the ASD and control groups.

As shown in Figure 6, the average ROC curve presents over 10-fold cross-validation. The red line represents the mean ROC across all folds, and its high and smooth shape indicates consistently strong performance in every validation split. This result demonstrates that DAGMNet achieves not only high prediction accuracy but also excellent robustness and generalizability across different data partitions.

### 3.4. t-SNE Two-Dimensional Visualization

**Figure 7:**
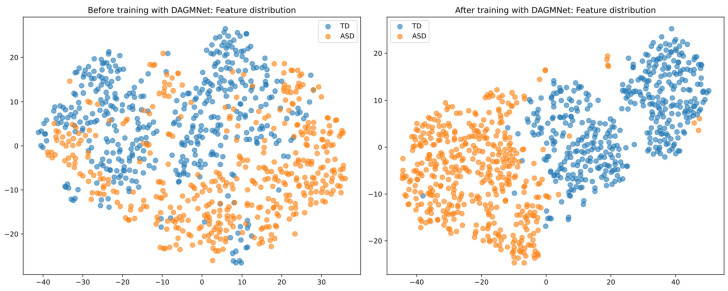
t-SNE visualisation of graph embeddings. (**Left**) Feature distribution before learning with DAGMNet shows significant overlap and dispersion between the ASD and control groups. (**Right**) After DAGMNet training, the learned features form distinct and compact clusters, demonstrating improved class separability.

As shown in Figure 7, before DAGMNet training, the data points are widely scattered, with considerable overlap between the visualisation of ASD and control groups, reflecting poor discriminative structure. However, after training, the learned representations are well-separated and exhibit clear clustering patterns, indicating that DAGMNet effectively captures meaningful and modality-integrated features.

### 3.5. Biomarker Detection

**Figure 8:**
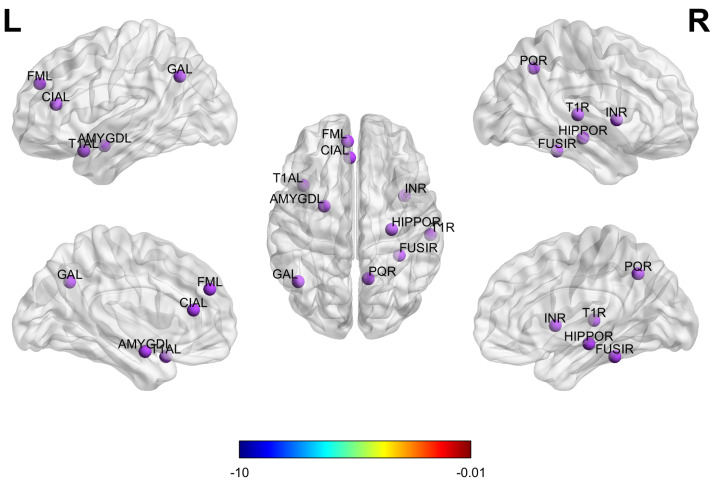
Ten brain regions most important for ASD prediction on ABIDE.

To identify the most discriminative brain regions for the diagnosis of ASD, this study utilizes the ESPM, which adaptively selects nodes from multi-atlas brain networks. During model training, the module suppresses less informative nodes and prunes weak connections while preserving and reweighting the most discriminative ones to highlight key information. The node importance coefficients (α) outputs by the ESPM are aggregated and ranked by their average scores so that the top 10 regions of interest (ROIs) can be selected as candidate neuroimaging biomarkers. The 10 brain regions most important for ASD classification are presented in Figure 8, which was generated using the BrainNet Viewer toolbox [29]. Functionally, these ROIs encompass multiple brain networks. The amygdala and hippocampus are involved in emotional regulation and memory processing [12]; the fusiform gyrus, superior temporal gyrus, and temporal pole are associated with social perception and face recognition; the insula is related to emotional processing [30]; the anterior cingulate cortex and medial superior frontal gyrus are implicated in cognitive control and self-referential processing; and the precuneus and angular gyrus serve as core nodes of the default mode network supporting social cognition and memory retrieval. The key brain regions identified in this study align closely with abnormal regions reported in multiple ASD neuroimaging studies [26,27,31], which further validates the reliability and scientific value of the proposed biomarker detection method.

## 4. Discussion

We propose DAGMNet, a novel graph neural network framework that integrates sMRI and fMRI data through modality-aware fusion and dynamic graph learning. Due to the preprocessing step in which features are extracted and graphs are constructed prior to multimodal feature fusion, the inference stage of the final GCN module is highly efficient, taking approximately 0.021 s per subject. The framework models both individual-level structural–functional interactions and cross-subject phenotypic similarity. Its architecture comprises three main components: (1) a representation learning module that adaptively captures shared and modality-specific features, (2) an edge-sensitive pooling strategy for multi-atlas topological refinement, and (3) a dynamic graph pruning mechanism to alleviate oversmoothing in GNNs. Experimental results on the ABIDE dataset show that DAGMNet outperforms existing baselines. Ablation experiments confirm the contribution of each module to robustness and predictive performance.

To evaluate generalization, DAGMNet is trained on ABIDE-I and tested on ADNI as an external cohort. This dataset includes different imaging sites and acquisition protocols. As shown in Table 6, the model achieved 87.09% accuracy and 72.66% AUC, demonstrating consistent performance across various conditions. However, ABIDE-I and ADNI represent specific demographic and technical contexts. Broader validation on diverse, large-scale, multi-site datasets is necessary before concluding full generalizability.

The current implementation relies on predefined anatomical atlases (HO, EZ, AAL) for graph construction. Although these atlases improve reproducibility and comparability, they cannot fully represent subject-specific anatomical variation or fine-scale functional boundaries. Data-driven or personalized parcellation schemes, including connectivity-based or adaptive multi-resolution approaches, may enhance sensitivity to subtle alterations. Moreover, validation has been limited to ABIDE-I and ADNI. Future studies should incorporate datasets that cover a wider range of ages, conditions, and scanner types. Domain adaptation and harmonization techniques could further improve cross-site transferability.

Finally, the present model incorporates sMRI, fMRI, and phenotypic variables. Expanding to additional modalities such as diffusion MRI, PET, or genomic data could improve robustness. Incorporating uncertainty estimation would provide clinically actionable confidence measures. Advanced interpretability tools, for example, attention-based saliency mapping or counterfactual reasoning, may also facilitate adoption in clinical workflows.

In future work, we aim to extend DAGMNet by integrating additional modalities and incorporating uncertainty estimation to enhance robustness. We also plan to explore domain adaptation techniques to improve cross-site transferability. Furthermore, embedding advanced interpretability tools, such as attention-based saliency maps or counterfactual reasoning, may facilitate clinical adoption. Finally, we intend to evaluate the model on large-scale, multi-site datasets to assess its scalability and real-world applicability.

## 5. Conclusions

In this paper, we propose DAGMNet, a novel dual-branch attention-pruned graph neural network for a multimodal network, aiming to accurately predict ASD. DAGMNet leverages both sMRI and fMRI data, employing modality-specific encoders and edge-sensitive pooling to extract hierarchical spatial and topological features effectively. Multi-atlas graph embeddings are integrated with imaging features through an attention-based multimodal fusion module, while a dynamic graph pruning mechanism enables individualized population-level reasoning. Our method achieves an AUC of 96.80% and an accuracy of 91.59% on the ABIDE dataset, outperforming several state-of-the-art baselines and demonstrating the effectiveness of our unified graph-guided framework for robust and interpretable ASD diagnosis.

## Figures and Tables

**Table 1:** Comparison with State-of-the-Art (SOTA) Models.

Method	sMRI	fMRI	Phenotype	ACC (%)	SEN (%)	SPE (%)	AUC (%)
DeepASD [13]	×	✔	✔	87.38 ± 2.87	88.35 ± 6.83	86.51 ± 8.41	92.76 ± 4.00
LG-GNN [12]	×	✔	✔	81.75 ± 1.10	83.22 ± 1.84	80.99 ± 1.17	85.22 ± 1.01
METAformer [21]	×	✔	✔	83.70 ± 0.53	90.10 ± 0.27	81.90 ± 0.12	83.20 ± 0.03
EV-GCN [17]	×	✔	✔	85.90 ± 4.47	88.23 ± 7.18	79.90 ± 7.37	84.72 ± 4.27
MMGL [23]	×	✔	✔	89.77 ± 2.72	90.32 ± 4.21	89.81 ± 2.56	89.30 ± 6.04
WL-DeepGCN [24]	×	✔	✔	77.27 ± 1.59	80.96 ± 2.57	77.70 ± 4.08	82.59 ± 2.80
BPGLNet [25]	×	✔	✔	86.45 ± 0.62	86.71 ± 0.22	87.14 ± 1.08	90.75 ± 0.17
GCN [22]	✔	✔	✔	81.23 ± 0.53	81.36 ± 0.30	81.02 ± 0.21	81.29 ± 0.13
MMKGL [26]	✔	✔	✔	91.08 ± 0.59	91.97 ± 0.64	90.05 ± 1.37	91.01 ± 0.63
MTGWNN [27]	×	✔	✔	87.25 ± 1.45	87.36 ± 2.49	87.75 ± 2.47	92.49 ± 1.55
DML-GNN [28]	×	✔	✔	90.93 ± 0.42	90.75 ± 0.24	92.74 ± 0.13	93.69 ± 0.12
**DAGMNet (Ours)**	✔	✔	✔	**91.59 ± 0.41**	**90.33 ± 0.12**	**93.23 ± 0.03**	**96.80 ± 0.02**

**Note:** “✔” indicates the modality is used in the method; “×” indicates the modality is not used. All metrics are reported as mean ± standard deviation across multiple runs.

**Table 2:** Ablation study of DAGMNet. Each variant removes one key component to assess its contribution to ASD prediction performance.

Method	ACC (%)	SEN (%)	SPE (%)	AUC (%)
w/o ESPM	87.63 ± 0.23	93.20 ± 0.83	90.11 ± 0.12	86.70 ± 0.06
w/o MARL	86.25 ± 0.11	90.85 ± 0.62	89.76 ± 0.21	85.93 ± 0.51
w/o PAPG	85.77 ± 0.21	89.12 ± 0.14	88.42 ± 0.34	84.65 ± 0.08
w/o ACFE	82.38 ± 0.32	86.07 ± 0.13	87.93 ± 0.04	82.21 ± 0.02
**DAGMNet (Ours)**	**91.59 ± 0.41**	**90.33 ± 0.12**	**93.23 ± 0.03**	**96.80 ± 0.02**

**Table 3:** Ablation study on the ACFE module. Each variant removes one key component to assess its contribution to ASD prediction performance.

Method	ACC (%)	SEN (%)	SPE (%)	AUC (%)
w/o 3DConv	83.20 ± 0.35	85.10 ± 0.40	82.50 ± 0.38	84.00 ± 0.30
w/o Channel Attention	87.10 ± 0.28	88.50 ± 0.25	86.30 ± 0.30	89.20 ± 0.25
w/o Spatial Attention	87.50 ± 0.30	88.00 ± 0.28	87.10 ± 0.33	89.80 ± 0.20
w/o Residual	89.00 ± 0.26	89.50 ± 0.22	88.80 ± 0.27	91.00 ± 0.18
**DAGMNet (ACFE)**	**91.59 ± 0.41**	**90.33 ± 0.12**	**93.23 ± 0.03**	**96.80 ± 0.02**

**Table 4:** Performance comparison of DAGMNet model variants using different modality inputs.

Method	ACC (%)	SEN (%)	SPE (%)	AUC (%)
DAGMNet (sMRI_img only)	86.79 ± 0.13	79.65 ± 0.19	92.86 ± 0.29	94.71 ± 0.31
DAGMNet (fMRI_img only)	85.77 ± 0.22	76.05 ± 0.43	94.49 ± 0.02	92.25 ± 0.21
HO atlas	89.15 ± 0.62	78.85 ± 0.75	88.68 ± 0.64	89.36 ± 0.47
EZ atlas	88.01 ± 0.45	77.35 ± 0.26	85.73 ± 0.73	86.93 ± 0.28
AAL atlas	87.03 ± 0.83	83.74 ± 0.36	86.82 ± 0.57	88.27 ± 0.37
multi-atlas only (HO, EZ, AAL)	91.15 ± 0.34	90.29 ± 0.67	91.93 ± 0.63	96.65 ± 0.33
**DAGMNet (Ours)**	**91.59 ± 0.41**	**90.33 ± 0.12**	**93.23 ± 0.03**	**96.80 ± 0.02**

**Table 5:** Comparison of different feature fusion methods on ASD prediction performance.

Multimodal Method	ACC (%)	SEN (%)	SPE (%)	AUC (%)
Concatenation	80.04 ± 0.12	59.63 ± 0.28	97.63 ± 0.22	94.73 ± 0.24
ECMA	83.23 ± 0.53	80.77 ± 0.65	85.23 ± 0.89	92.28 ± 0.05
Self-Attention	86.44 ± 0.76	81.28 ± 0.25	90.48 ± 0.76	95.58 ± 0.41
Cross-Attention	86.80 ± 0.11	86.55 ± 0.33	89.31 ± 0.34	95.33 ± 0.81
**DAGMNet (Ours)**	**91.59 ± 0.41**	**90.33 ± 0.12**	**93.23 ± 0.03**	**96.80 ± 0.02**

**Table 6:** Results of our method on the ADNI datasets.

Method	sMRI	fMRI	Phenotype	ACC (%)	SEN (%)	SPE (%)	AUC (%)
DeepASD [13]	×	✔	✔	80.73 ± 2.47	77.94 ± 3.56	58.38 ± 5.72	67.46 ± 2.55
LG-GNN [12]	×	✔	✔	82.09 ± 1.40	82.96 ± 0.91	51.66 ± 4.84	68.33 ± 3.47
METAformer [21]	×	✔	✔	79.45 ± 1.07	81.19 ± 0.45	60.42 ± 1.37	68.81 ± 0.95
EV-GCN [17]	×	✔	✔	80.42 ± 3.36	80.86 ± 4.70	62.56 ± 5.26	67.82 ± 3.86
MMGL [23]	×	✔	✔	84.65 ± 1.89	81.35 ± 3.47	63.54 ± 2.36	67.74 ± 5.28
WL-DeepGCN [24]	×	✔	✔	75.36 ± 1.45	74.53 ± 2.73	57.27 ± 3.15	63.20 ± 2.25
BPGLNet [25]	×	✔	✔	80.65 ± 0.82	77.28 ± 0.39	62.45 ± 1.45	66.23 ± 1.71
GCN [22]	✔	✔	✔	77.18 ± 0.68	72.46 ± 1.38	60.78 ± 0.57	63.53 ± 1.52
MMKGL [26]	✔	✔	✔	85.75 ± 0.63	82.55 ± 1.59	63.29 ± 1.39	69.16 ± 1.46
MTGWNN [27]	×	✔	✔	81.47 ± 1.56	78.28 ± 2.43	60.75 ± 2.64	65.77 ± 1.77
DML-GNN [28]	×	✔	✔	83.88 ± 0.58	80.23 ± 0.88	62.35 ± 1.25	69.20 ± 0.94
DAGMNet (Ours)	✔	✔	✔	87.09 ± 1.73	85.83 ± 0.76	65.92 ± 2.20	72.66 ± 2.65

**Note:** “✔” indicates the modality is used in the method; “×” indicates the modality is not used. All metrics are reported as mean ± standard deviation across multiple runs.

## Data Availability

This study utilizes the Autism Brain Imaging Data Exchange (ABIDE-I) dataset, a publicly available resource that provides multimodal neuroimaging data. The dataset can be accessed at: https://fcon_1000.projects.nitrc.org/indi/abide/abide_I.html, accessed on 1 September 2025.
